# Editorial of the Special Issue “Dietary Fiber and Inflammatory Bowel Disease”

**DOI:** 10.3390/nu14224861

**Published:** 2022-11-17

**Authors:** Gang Liu, Xiaoyue Xu

**Affiliations:** 1 School of Life Sciences, Faculty of Science, University of Technology Sydney, Ultimo, NSW 2007, Australia; 2 Centre for Inflammation, Centenary Institute, Camperdown, NSW 2050, Australia; 3 School of Population Health, University of New South Wales, Sydney, NSW 2052, Australia

Inflammatory bowel disease (IBD), including Crohn’s disease and ulcerative colitis, is a chronic disease of the gastrointestinal (GI) tract; its burden has significantly increased in recent decades, with 6.8 million cases of IBD reported in 2017 according to the Global Burden of Disease study [1]. The exact cause of IBD remains unknown, although chronic gut inflammation has been found to be a key contributing factor in the development of IBD, resulting in tissue remodelling that damages the structure of the colon. Current treatments only slow its progression, with no imminent cure in sight; however, surgical resection to physically remove damaged areas in the GI tract is still considered the best option for the treatment of IBD, potentially calling for multiple surgeries due to the recurrence of the disease [2]. Therefore, a better understanding of IBD development and elucidation of its underpinned mechanisms could help explore novel therapeutic options for this disease.

Mucosal inflammation, immune cell infiltration and increased leukocytes are regularly observed in IBD patients, always associated with immune system malfunctions, including T helper (Th)2 and Th17 responses and abnormal levels of cytokines [3]. For example, the tumour necrosis factor (TNF) is a proinflammatory cytokine whose level tends to significantly increase in IBD patients [4]. Chronic inflammation in patients with IBD also results in structural cells (epithelial cells, fibroblasts and muscle cells) to secrete extracellular matrix (ECM) proteins, including collagen and fibronectin for wound healing [5]. However, the uncontrolled wound healing process in patients leads to the extensive deposition of ECM proteins and tissue remodelling/fibrosis that damages the structure and function of colon tissue.

The gut microbiome is essential in regulating the immune system in IBD patients, with studies having shown varying microbial diversities in IBD patients and healthy individuals. The maintenance of the balance of the gut microbiota is a novel insight in the treatment of IBD [6]. For example, faecal microbiota transplantation (FMT) has recently become a novel therapeutic concept, where functional microbiota are transplanted from faeces donated by healthy donors to IBD patients, for which many clinical trials are currently in progress [7].

Interestingly, IBD patients have presented with similar microbial changes to patients with COVID-19, characterised by gut issues, including diarrhea, vomiting and abdominal pain. This could potentially indicate a link between COVID-19 and IBD, but recent studies have shown that IBD patients have no increased risk of contracting COVID-19 [8]. The relationship between IBD and COVID-19, including their underpinned mechanisms, requires further discussion.

The rapid ageing of the population is a global phenomenon that entails the elderly people to be more prone to developing IBD. Biological aging results in cellular senescence that leads to immune dysfunction and chronic inflammation [9], a process that also significantly contributes to the progression of IBD.

Diet has been widely recognised as an important factor in the prevention and management of noncommunicable diseases (NCDs) [10,11,12,13,14,15], including IBD. Several pathways where the diet might influence intestinal inflammation have been proposed thus far, including direct dietary antigens being responsible for altering the gut microbiome and affecting gastrointestinal permeability [16].

Accumulating epidemiological and experimental studies have reported that diets rich in fibre or particular dietary patterns (e.g., plant-based diets) might help in the management of IBD [17]. However, more research comprising strong and robust evidence in support of effective dietary interventions in achieving better clinical outcomes among patients with IBD is needed.

In this Special Issue, manuscripts highlight creative and novel findings linking diet and IBD and present research to help advance the knowledge of how dietary fibre affects IBD in biomedical science, the population’s health and clinical practice.

## Data Availability

Not applicable.
